# Novel Approaches to Allergen Immunotherapy for Respiratory Allergies

**DOI:** 10.3390/ph17111510

**Published:** 2024-11-09

**Authors:** Mongkol Lao-Araya

**Affiliations:** Division of Allergy and Clinical Immunology, Department of Pediatrics, Faculty of Medicine, Chiang Mai University, Chiang Mai 50200, Thailand; laoaraya@gmail.com

**Keywords:** allergy, allergen immunotherapy, allergic rhinitis, modified allergen, biologics, adjuvant

## Abstract

Allergen immunotherapy (AIT) remains the cornerstone for managing respiratory allergies, offering long-term symptom relief, disease modification, and prevention of disease progression. While novel approaches like intralymphatic and epicutaneous immunotherapy and the combination of allergens with adjuvants show promise, traditional methods remain effective and safe. Hypoallergenic T-cell peptide vaccines and recombinant allergens require further research to confirm their clinical benefits. Passive immunotherapy, while demonstrating effectiveness in specific cases, needs exploration of its long-term efficacy and broader applicability. Combining AIT with biologics may enhance safety and treatment outcomes. Despite emerging innovations, allergen-specific immunotherapy with natural allergen extracts remains the primary disease-modifying treatment, offering long-term symptom relief and prevention of disease progression. Continued research is essential to refine and optimize allergen immunotherapy strategies, providing patients with more effective and personalized treatment options.

## 1. Introduction

Allergen immunotherapy (AIT), a therapeutic approach rooted in the concept of immunization, has evolved significantly since its inception. Its origins can be traced back to Edward Jenner’s pioneering smallpox vaccine in 1796, demonstrating the therapeutic potential of inducing immunity. The modern understanding of allergic disease emerged in the late 19th century with Blackley’s observations linking pollen exposure to hay fever symptoms [1,2]. The therapeutic application of AIT for hay fever was first introduced by Noon and Freeman in 1911, marking the beginning of a century-long journey of research and development [3]. While early studies laid the groundwork, rigorous clinical trials with well-characterized allergen extracts did not emerge until the 1980s, establishing the dose-dependent therapeutic effect of AIT [2,4,5,6,7].

Over the past century, AIT has undergone significant advancements. Despite its initial discovery over 110 years ago, AIT remains the standard therapy for allergic rhinitis (AR) and asthma [1,2]. Its proven efficacy in reducing symptoms and improving quality of life has earned the recommendation of numerous medical organizations [8,9,10]. AIT offers a unique disease-modifying approach, inducing long-term allergen tolerance and reducing allergic inflammation [11]. This is achieved by administering increasing doses of allergens via subcutaneous (SCIT) or sublingual (SLIT) routes.

While AIT offers substantial benefits, traditional treatment regimens can be lengthy and associated with adverse reactions. The need for prolonged administration, often spanning three years or more, can deter patients. Additionally, the risk of adverse reactions, particularly during dose escalation, can lead to treatment discontinuation [12]. Furthermore, AIT’s effectiveness varies across different allergens and patient populations [13].

**Figure 1:**
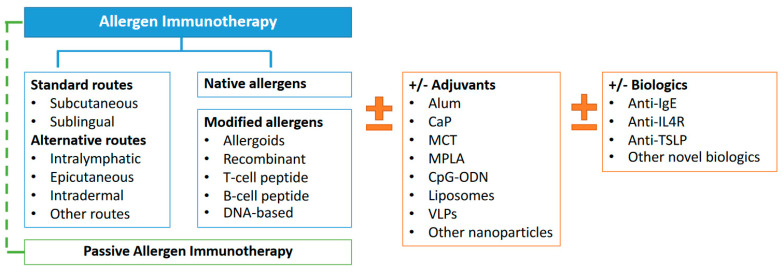
Approaches of allergen immunotherapy for respiratory allergies.

To address these limitations, ongoing research focuses on optimizing AIT delivery methods, treatment procedures, and patient adherence, particularly in pediatric populations. Novel approaches, such as allergen component therapy, hypoallergenic immunotherapy, new delivery routes, and the combination of allergens with adjuvants or biologics, hold promise in enhancing the efficacy and safety of AIT [2,14,15,16,17,18]. These innovations are expanding the therapeutic landscape for AIT, offering hope for individuals with respiratory allergies (Figure 1).

This review will explore the latest advancements in AIT, encompassing improvements in allergen extracts, delivery methods, and treatment strategies. By highlighting these innovations, we aim to provide insights into the future of AIT and its potential to enhance the lives of individuals with respiratory allergies.

## 2. Mechanisms of AIT (Table 1)

The allergic response is a multifaceted process involving the interaction of various immune cells, mediators, and cytokines. It begins when antigen-presenting cells (APCs), such as dendritic cells (DCs), recognize an allergen. These APCs, activated by epithelial-derived cytokines and those from type 2 innate lymphoid cells (ILC2) [19] and basophils, present the allergen to naive T cells, guiding their differentiation into T-helper 2 (TH2) cells. TH2 and follicular T-helper (Tfh) cells play a pivotal role in driving the allergic response by producing IL-4 and IL-13. These cytokines promote IgE production by B cells, enhancing the allergic inflammatory environment. In individuals with allergies, repeated exposure to low-dose allergens through a compromised epithelial barrier can lead to increased sIgE levels and subsequent allergic reactions [20,21,22].

While research on AIT mechanisms has primarily focused on aeroallergens, studies suggest that high-dose allergen exposure can play a pivotal role in restoring the epithelial barrier and inducing a shift from allergic TH2 inflammation to a more tolerant TH1 response [23]. This shift involves the generation of suppressive regulatory immune cells and a decrease in pro-allergic cytokines (IL-4, IL-5, IL-9, and IL-13). The reduction in allergic inflammation is accompanied by a decrease in mast cells, basophils, and eosinophils, key cells involved in allergic reactions. Additionally, there is an increase in allergen-specific T regulatory (Treg) cells [24,25], T follicular regulatory (Tfr) cells [26], and B regulatory (Breg) cells, which can help suppress the allergic immune response [22,23].

Furthermore, AIT is effective in inducing dendritic cell-derived regulatory cells (DCregs) [27] and innate lymphoid cell-derived regulatory cells (ILCregs) [28,29,30]. These regulatory cells produce cytokines like TGF-β, IL-12, IL-27, and IL-10, which can suppress allergic responses [24,31]. AIT is also associated with the generation of Treg-cell subsets, which can further suppress TH2 and Tfh-cell responses, leading to a shift towards TH1 cells [32].

Initial immunotherapy may lead to a temporary increase in sIgE levels. However, over time, the immune response shifts towards a tolerogenic state. Breg cells, stimulated by cytokines like IL-10 and IL-35, play a crucial role in this process. These cells promote the production of blocking antibodies, including IgG1, IgG4, IgA1, and IgA2, which can bind to allergens and prevent them from triggering allergic responses [33]. These blocking antibodies are found both in the bloodstream and in mucosal secretions. For SCIT, IgG4 is the primary blocking antibody, while for SLIT, IgA1 and IgA2 are more prominent in both nasal and systemic compartments [23,34,35,36].

In summary, the mechanisms of immunotherapy involve a complex interplay of innate and adaptive immune cells, cytokines, and antibodies, ultimately leading to a shift towards a more tolerant immune response and reduced allergic symptoms [23,37].

**Table 1:** A summary of the main mechanisms and proven clinical benefits of allergen immunotherapy.

Mechanism of AIT
Restoration of epithelial cell integrity Decrease in allergen-dependent mast cell/basophil degranulation Reduction in type 2 immune responses Regulation of T-cell responses: suppression of TH2 and immune deviation toward a TH1 response, reduction of circulating Tfh cells Modulation of ILCs: reduction of circulating ILC2s, induction of IL-10+ regulatory ILC2s Induction of regulatory response: induction of Treg and Breg cells Stimulation of allergen-specific blocking antibody production, including IgG1, IgG2, IgG4, and IgA, in both systemic and mucosal immune responses Induction of tolerogenic cytokines: IL-10, IL-12, IL-27, IL-35 and TGF-β
**Proven clinical benefits of AIT in allergic rhinoconjunctivitis +/− asthma**
Reduced symptoms and medication use Improved quality of life The benefits can last for many years, even after treatment is stopped. Reduced risk of developing asthma in children with AR Prevent subsequent allergic sensitization

## 3. Conventional AIT: SCIT and SLIT

SCIT and SLIT have been used as standard treatments for respiratory allergies for decades [10]. SCIT involves administering allergen extracts in gradually increasing doses over several weeks or months, followed by a maintenance phase of monthly injections for 3 to 5 years. While SCIT is generally safe, it carries a risk of systemic allergic reactions (up to 22%), including anaphylaxis [8,10,38,39,40]. However, the risk can be minimized with appropriate patient selection, adequate facilities, well-trained staff, and availability of emergency treatment [8,10]. The efficacy of SCIT varies depending on the allergen and the specific product used. Meta-analyses have demonstrated that SCIT is approximately 30% more effective than placebo in treating seasonal and perennial allergic rhinitis, [41] exceeding the World Allergy Organization’s defined minimally clinically important difference of 20% [42].

While SCIT has been shown to be as effective as or more effective than pharmacotherapy in extrapolated pooled analyses, there are few direct comparisons between the two [43,44]. In practice, adherence and persistence with SCIT can be challenging, with studies showing that less than 50% of users completed the recommended 3 years of treatment [12,45].

In recent years, SLIT has emerged as a well-validated alternative to SCIT. Several large randomized controlled trials have confirmed the efficacy of SLIT tablets for patients with house dust mite (HDM), [46,47,48] grass pollen, [49] ragweed, [50] and Japanese cedar [51,52] pollen allergic rhinitis, including those with mild to moderately severe controlled HDM-induced allergic asthma [53,54].

SLIT involves taking tablets or drops sublingually daily for 3 years or starting 2 to 4 months before the allergy season in patients with seasonal AR [55]. After initial supervision and a 30-min observation period, SLIT can be self-administered, making it convenient for patients. Home administration and scheduled follow-up visits contribute to improved adherence with SLIT. Compared to SCIT, SLIT is generally safer. While SLIT is associated with local side effects like oropharyngeal itching and swelling, these are typically self-limiting and resolve within 1 to 2 weeks. Systemic side effects are rare, making SLIT a safer alternative to SCIT [38,56,57].

Both SCIT and SLIT have been shown to be effective for both seasonal and perennial allergies [41]. Indirect meta-analyses suggest that SLIT is at least as effective as current pharmacotherapy, although head-to-head controlled studies are needed for definitive confirmation [44,58]. A recent direct comparison using nasal allergen challenge found that SCIT was more effective than SLIT in the first year of treatment. However, the two treatments were equally effective in the second year [36]. Due to the heterogeneity between studies, treatment decisions should be based on the evidence available for specific products and individual patient factors [57,59,60].

## 4. Alternative Routes (Table 2)

### 4.1. Intralymphatic Immunotherapy

Intralymphatic immunotherapy (ILIT) is a targeted immunotherapy approach that involves injecting allergen extracts directly into lymph nodes, usually in the groin, under ultrasound guidance [61]. An ILIT injection of recombinant allergens (phospholipase A2 and Fel d 1) has demonstrated a significant increase in IgG levels compared to SCIT, achieving a 10-fold increase in just two weeks at a 100-fold lower dose [62,63]. This direct approach targets the immune system, potentially enhancing allergen presentation to T cells and avoiding direct mast cell activation [18,64].

A randomized open-label trial compared a 3-dose ILIT regimen administered over 2 months to a 3-year SCIT regimen in patients with grass pollen allergy, finding that ILIT achieved a persistent effect over 3 years with fewer side effects and significantly improved compliance [61]. While ILIT resulted in slightly less medication use and similar symptom score improvements, the low compliance rate in the SCIT group and the open-label nature of the study make it challenging to draw definitive conclusions.

Several studies have shown the efficacy of ILIT for grass and tree pollen allergy, although not all studies have confirmed its effectiveness [61,65,66]. A small-scale study showed that three intralymphatic injections of a recombinant cat Fel d 1 allergen, fused with a translocation sequence and a human invariant chain fragment, were effective in protecting against nasal challenge with whole cat allergen extract [62].

Overall, ILIT offers potential advantages in terms of reduced side effects, improved compliance, and shorter treatment duration [61,67]. However, it requires specialist skills, experience, and ultrasound guidance for injections, making it more technically demanding than traditional routes. Individual products, doses, and timing of injections still need to be optimized [64]. Further research is needed to confirm its efficacy and safety for various allergens and patient populations.

**Table 2:** Routes of allergen immunotherapy administration.

Routes of AIT	Advantages	Drawbacks
Subcutaneous (SCIT)	Proven efficacy and safety Standard applications in respiratory and venom AIT	Repeated injections Healthcare unit dependency Long duration of treatment Risk of severe hypersensitivity reactions
Sublingual (SLIT)	Proven efficacy and safety Standard applications in respiratory AIT No injections Less healthcare unit dependency Home application Lower risk of severe hypersensitivity reactions	Need for daily self-applications which may affect the adherence Higher allergen dose
Intralymphatic (ILIT)	Applicability in respiratory and venom allergens Reduced number of injections Reduced treatment duration Reduced allergen dosage use Early clinical studies revealed promising results	Requirement of experienced HCP for injections under ultrasound guidance Risk for side effects due to allergens injected into lymph nodes Further clinical study needed
Epicutaneous (EPIT)	Applicability in food and respiratory allergens Good safety profile Early clinical studies revealed promising results	Risk of local adverse reactions Further clinical study needed
Intradermal (IDIT)	May reduce allergen dosage use	No clinical efficacy proven

### 4.2. Epicutaneous Immunotherapy

Epicutaneous immunotherapy (EPIT) involves applying allergen patches to the skin for several hours, aiming to increase local antigen presentation while preventing systemic allergen absorption. By removing the top layer of skin with adhesive tape, keratinocytes can be activated, increasing allergen exposure and potentially stimulating DC responses. This needle-free approach can improve patient compliance, especially in children [68,69].

A placebo-controlled randomized trial applied grass pollen extract in petroleum to the skin, stripped with adhesive tape, weekly for 6 months pre-seasonally in 48 participants. The study observed a 48% improvement in seasonal symptom scores in the first year compared to a 10% improvement in the placebo group. However, there were no significant differences in combined treatment and medication scores. Two further randomized controlled trials achieved similar results [70]. While higher doses were effective, they also led to high rates of local irritation, eczema, and occasional systemic allergic side effects, limiting their clinical utility compared to currently available SCIT [17,71].

A placebo-controlled randomized trial applied grass pollen extract to the skin, prepared with adhesive tape, weekly for 6 months pre-seasonally in 48 participants. The study observed a significant improvement in seasonal symptom scores in the treatment group compared to the placebo group [70]. However, while higher doses were effective, they also led to increased local skin reactions and systemic side effects, limiting their clinical utility. Two further randomized controlled trials yielded similar results. While EPIT has shown promise, its efficacy and safety profile still need to be optimized to compete with established SCIT [17,71].

### 4.3. Intradermal Immunotherapy

Intradermal allergen administration may increase the immune response and decrease the required allergen dose due to the presence of DCs in the intradermal area [17,71]. Early studies suggested that repeated low-dose intradermal injections could suppress late allergic responses and induce allergen-specific IgG antibodies [17,72]. However, a phase 2b trial of pre-seasonal low-dose intradermal grass pollen allergen failed to demonstrate significant clinical improvement. In fact, nasal symptoms worsened, and a heightened TH2 response was observed at the injection site [73]. These findings suggest that intradermal allergen administration may have paradoxical effects, potentially inducing both sensitization and tolerance. Therefore, this approach is not currently recommended for allergen immunotherapy.

## 5. Adjuvants (Table 3)

An adjuvant is a substance that enhances the immune response to a vaccine. By physically or chemically interacting with antigens, adjuvants can modify the pharmacological and immunological effects of allergen vaccines. They can modulate allergen delivery, act as a depot, stimulate immune responses, and steer the immune response towards either tolerance or a TH1-biased response. Additionally, adjuvants can help reduce the risk of anaphylactic reactions and unwanted side effects [14,17,18,74].

Traditionally, adjuvants have been classified into first-generation (aluminum hydroxide, microcrystalline tyrosine [MCT], and calcium phosphate) and second-generation (Monophosphoryl Lipid A [MPLA], Toll-like receptor [TLR] agonists) categories. Other promising adjuvants include liposomes and virus-like particles (VLP) [14]. The adjuvants currently approved for use in humans (aluminum hydroxide, calcium phosphate, MCT, and MPLA) [18] offer several advantages for improving AIT. These include prolonging antigen exposure at the injection site and stimulating the production of allergen-specific IgG antibodies.

**Table 3:** Adjuvants in allergen immunotherapy.

Adjuvants	Approved for Clinical Use [18]	Mechanism of Action
Aluminum hydroxide (alum)	EU (1937)	Allergen depot Inhibits TH2 and enhances TH1 responses Low biodegradability, may lead to adverse reactions
Calcium Phosphate (CaP)	EU (1980)	Allergen depot Natural component High biodegradability
Microcrystalline tyrosine (MCT)	EU (1970)	Biodegradable depot adjuvant Inhibits TH2 and enhances TH1 responses
Monophosphoryl Lipid A (MPLA)	EU (1999)	TLR-4 agonist Stimulates TH1 response Works synergistically with MCT
CpG-Oligodesoxy- nucleotides	No	TLR-9 agonist Stimulates TH1 response Combined in VLP as ‘allergen-independent TH1 simulant’ Conflicting results in Phase 3 clinical trial
Nanoparticles: lipophilic liposomes, virus-like particles (VLPs) and other particles from synthetic and natural polymers	No	Encapsulation of allergen Allowing uptake into APCs without IgE binding Activation of innate immunity without T cell help Limited data in clinical trials

### 5.1. Alum and Calcium Phosphate

Aluminum salts (alum) have been used as a vaccine adjuvant since 1926 [75] and remain one of the most widely used adjuvants in human vaccines [74]. While alum-based allergen extracts are licensed for SCIT in Europe, they are not approved for use in the United States. Alum functions by adsorbing the allergen and triggering both innate and adaptive immune responses, including inflammasome activation and T-cell activation, which can enhance antibody responses. Although alum can increase TH2 responses in mouse models during sensitization, in humans, it has been shown to inhibit established allergic TH2 responses and promote TH1 responses, both in vitro and in vivo [14,74].

Alum-based allergy extracts have a long history of safe and effective use in Europe. While alum is well-tolerated, it can induce acute and chronic inflammation at the injection site due to its low biodegradability. Although there is a theoretical risk of aluminum accumulation and systemic side effects, this has not been observed in humans [74,76].

Calcium phosphate, a natural component of the body, is another depot adjuvant with better biodegradability and biocompatibility than alum [14]. Although calcium phosphate-adjuvanted AIT products were once available in the European market, they are no longer available for reasons that remain unclear [74].

### 5.2. Microcrystalline Tyrosine (MCT)

MCT, the crystalline form of the non-essential amino acid L-tyrosine, is a promising adjuvant for AIT [14,74]. While less commonly used than alum, MCT has demonstrated safety and efficacy in humans. It is a biodegradable depot adjuvant with a short half-life of 48 h. Since its initial report in the 1980s, MCT has been shown to enhance the induction of IgG antibodies when used with allergenic molecules. Unlike aluminum, which can accumulate at injection sites in murine models of AIT and is associated with granuloma formation, MCT is rapidly released and metabolized, reducing the risk of long-term accumulation [77,78].

MCT, a non-toxic adjuvant except in individuals with tyrosine metabolism disorders, [14] is currently patented for immunotherapy. It is integrated into glutaraldehyde allergoids to alleviate allergic symptoms and reduce reliance on relief medications [79].

### 5.3. Toll-like Receptors (TLRs)

Toll-like receptors (TLRs), a class of pattern-recognition receptors, are primarily expressed on APCs. Upon recognition of specific pathogen-associated molecular patterns (PAMPs), they initiate both innate and adaptive immune responses [14,17,74].

Monophosphoryl lipid A (MPL), a TLR4 agonist derived from the lipopolysaccharide of *Salmonella minnesota*, stimulates the production of IFN-γ and IL-12 but does not promote IL-5 synthesis [74]. MPL has shown promise as an adjuvant for AIT. In a study of grass pollen allergy, pre-seasonal injections with MPL-containing allergoids led to a significant reduction in symptoms compared to placebo, with effects lasting up to 5 years after discontinuation [80,81].

MCT-adjuvanted allergen vaccines, which may include native or modified (allergoid) allergens, are often combined with MPL, as exemplified by Pollinex^®^Quattro, an allergen therapeutic available for the treatment of seasonal allergic rhinoconjunctivitis. Combining allergens with MPL and MCT may address some of the major drawbacks of traditional AIT, including long treatment durations, poor patient adherence, and adverse effects [82,83,84,85]. While Pollinex^®^Quattro is currently available in Germany, sufficient clinical human data supporting marketing authorization in other European states have not been reported [74].

Cytosine-phosphodiester-guanine (CpG) motifs, conserved PAMPs-bacterial DNA sequences, are recognized by TLR-9, a receptor primarily expressed on B cells and plasmacytoid dendritic cells (pDCs). Activation of TLR-9 stimulates the innate immune response in a TH1-type fashion [18,74]. A phase 2 trial demonstrated that combining CpG motifs with the ragweed allergen Amb a 1 suppressed seasonal symptoms in ragweed allergy patients [86]. However, these results were not replicated in a larger phase 3 trial [17]. While TLR-9 agonists show potential as adjuvants for AIT, additional research is needed to fully elucidate the mechanisms of action of these adjuvants and optimize their clinical application.

### 5.4. Liposomes and Virus-like Particles (VLPs)

Polymeric biodegradable nanoparticles, including polyesters, polysaccharides, polyamides, liposomes, and virus-like particles (VLPs), offer promising strategies for encapsulating allergens or proteins and delivering them to APCs without IgE binding. While many of these delivery systems are still under development, they have shown potential for inducing TH1-biased immune responses in preclinical studies [14,17,74].

Liposomes, spherical or vesicular structures composed of lipids, can effectively package and deliver water-soluble antigens to target cells. Studies have demonstrated that liposome-encapsulated allergens can induce higher levels of allergen-specific IgG while reducing IgE production in mice [87]. Liposomes may also enhance lymph node allergen delivery, potentially improving the efficacy of AIT [64].

VLPs, derived from viral capsids, can activate the immune system through innate pathways, bypassing the need for T-cell help. VLPs are efficiently taken up by APCs, resulting in the activation of cytotoxic T cells and the complement system. VLPs can be used in various ways for AIT, including as standalone VLPs with or without embedded adjuvants, as solutions mixed with allergens, or with allergens encapsulated within the VLPs. These different approaches offer flexibility in designing AIT regimens and may have varying effects on the immune response. Conjugating allergens with VLPs can enhance their uptake into the lymphatic system, potentially minimizing the risk of mast cell degranulation [2,16,74].

VLPs have demonstrated promising results in preclinical studies. A phase 2 trial involving patients with HDM allergy found that combining VLPs with a cytosine-phosphodiester-guanine TLR-9 agonist (CβG10) and allergen encapsulation (CYT003) resulted in similar symptom improvements compared to the adjuvant alone [88,89]. Encouraged by these findings, larger placebo-controlled trials were conducted. A double-blind, randomized trial involving 299 HDM allergy patients found that QβG10, a VLP-based therapy administered without additional allergen, reduced symptom scores in a dose-dependent manner [90]. Additionally, a higher dose of QβG10 was associated with a greater number of patients achieving increased allergen tolerance [90]. These results suggest that VLP-based therapies, even without the addition of allergens, may have therapeutic benefits in allergic diseases [16,74].

However, subsequent trials yielded mixed results. A study in asthmatic patients undergoing steroid withdrawal found that QβG10 reduced asthma-related symptom scores and increased the number of patients with well-controlled asthma [88]. However, a larger phase IIb trial in patients with persistent moderate-to-severe allergic asthma did not demonstrate significant differences between QβG10 and placebo [91].

Nanoparticle-based delivery systems offer potential advantages for AIT by improving allergen delivery, modulating immune responses, and potentially reducing side effects. However, further research is necessary to evaluate their safety and efficacy in clinical trials.

## 6. Modified Allergens (Table 4)

Modifying allergens to alter their tertiary protein structure or target non-IgE-reactive epitopes can help preserve or enhance their ability to induce immune responses while minimizing allergic reactions, promoting tolerogenic outcomes.

**Table 4:** Modified allergens and novel approaches in allergen immunotherapy.

Modified Allergens	Advantages	Drawbacks
Allergoids	Reduced allergenicity, improved safety profiles, shorter up-dosing phase	Limited efficacy data compared to conventional allergen extracts
Recombinant allergens	Personalized AIT, almost unlimited supply, may improve safety and efficacy	Limited efficacy and safety advantages compared to conventional extracts, reported late-phase adverse allergic reactions
T-cell peptides	Retained T-cell epitope stimulation, reduced IgE binding, may improve safety and efficacy	Potential for late-phase adverse responses, similar side effects to SCIT, failure to demonstrate clinical efficacy in phase 3 trials
B-cell peptides	Induce protective humoral antibody responses without stimulating IgE production, demonstrated increases in blocking IgG1 and IgG4 antibodies in phase 2 trial	Limited clinical data, did not reach statistical significance in primary analysis of combined seasonal symptom medication scores
DNA-based vaccine	Induce TH1 and Treg cell responses while downregulating TH2 cell responses in preclinical studies	Concern of theoretical risks of plasmid DNA integration into the human genome and development of anti-DNA antibodies, failure to demonstrate clinical efficacy in phase 3 trials
**Passive immunotherapy (monoclonal antibodies)**	Protection against nasal allergen challenge for almost 3 months after 1 dose injection	No long-term clinical efficacy shown
**AIT combined with biologics**	Enhanced efficacy and safety, potential for long-term allergen tolerance	High cost, further research needed to evaluate long-term benefits

### 6.1. Allergoids

Allergoids are chemically modified allergens produced through polymerization with glutaraldehyde or formaldehyde or monomerization with carbamylation. These modifications aim to alter the tertiary protein structure, reducing the ability of allergens to cross-link IgE while preserving shorter linear T-cell epitopes, thereby retaining immunogenicity. This approach allows for higher doses to be administered during a shorter-term accumulation phase, potentially improving treatment efficiency and reducing the risk of side effects [14,18,92].

Several allergoids have demonstrated efficacy in placebo-controlled trials for various allergens, including ragweed [92,93], grass [94,95,96], tree pollen [97,98,99], and mite [100,101,102,103,104] allergy. A phase 3 trial of a formaldehyde-treated, alum-adsorbed 6-grass pollen mix showed a significant 26.6% reduction in combined seasonal symptom medication scores after one year compared to placebo. Although some participants experienced mild to moderate late systemic reactions, no serious adverse events, such as anaphylaxis, were reported [105].

Carbamylation, a chemical modification of the lysine groups, has been used to develop low molecular weight allergoids that can be easily absorbed by the mucosa. A sublingual allergoid produced using this method reduced the need for antihistamines during the pollination season and maintained its clinical benefits for at least two years [106,107]. Another example is the HDM-monomeric allergoid SLIT, which has shown promise in improving rhinitis severity and reducing drug intake in phase II research [102,104]. These findings suggest that allergoids may offer a viable treatment option for respiratory allergies, with potential advantages over traditional allergen extracts.

Head-to-head comparisons between allergoids and conventional allergen extracts are limited, making it difficult to directly assess their relative efficacy. Quantification of modified allergens can be complex, leading to batch-to-batch variation and hindering comparisons of hypoallergenic effects between allergoids and whole allergens, both in vitro and in vivo [17,108].

### 6.2. Recombinant Allergens

Molecular diagnostics and therapeutics have significantly advanced the field of allergy medicine, enabling the identification of individual allergic components. This has paved the way for more precise allergy diagnosis and treatment, including the potential for personalized “tailor-made” allergen immunotherapy [109,110,111].

Molecular diagnosis also helps assess the clinical relevance of allergen sensitization profiles, particularly in cases of cross-reactivity between pollens and certain foods or different pollen types. By differentiating between true significant sensitization and pan-allergen sensitization, clinicians can select the most appropriate allergens for inclusion in immunotherapy, optimizing treatment outcomes for patients with complex sensitization profiles [109,110,111].

The potential of recombinant allergens to improve the safety and efficacy of AIT has been explored. The first cloning of allergen cDNAs was attempted in the late 1980s, and the first clinical trial using recombinant hypoallergens was conducted in the early 2000s [16,112,113].

A phase 2 trial showed that a mixture of six-grass pollen recombinant allergens was effective in treating seasonal allergic rhinitis. This treatment was associated with increases in allergen-specific IgG but not specific IgE, unlike whole allergen extract immunotherapy [114]. A phase 3 randomized controlled trial of recombinant birch allergen Bet v 1 demonstrated a 50% improvement in medication and symptom scores in patients with seasonal allergic rhinitis. While effective, it did not show significant differences in efficacy or side effects compared to natural protein or whole Bet v 1 extract [115].

Recombinant allergens offer several advantages, including standardization, genetic modification to deliver specific IgE-binding and T-cell epitopes, and an almost unlimited supply of purified allergen [14,16]. However, there are also potential disadvantages. One limitation of recombinant proteins is that they expose patients to only one or a few allergen molecules, unlike natural extracts that contain multiple allergens. While targeting a single allergen component may be effective for some patients, those sensitized to multiple components may benefit less from this approach. Additionally, recombinant allergens were initially thought to be safer due to their potential to stimulate only T or B cells, avoiding mast cell activation and related adverse events. However, clinical trials have reported adverse allergic reactions, including anaphylaxis, even hours after administration [16,112].

Despite showing promise in phase 2 trials, recombinant vaccines and hypoallergenic variants have not yet demonstrated significant improvements in efficacy or safety compared to current standardized allergen extracts. Further research is necessary to fully assess their potential benefits and address their limitations. With increasing knowledge of individual sensitization profiles and the role of major allergen epitopes, recombinant approaches may become more significant in the future of AIT [16].

### 6.3. T-Cell Peptides

T-cell epitopes are short, linear sequences of amino acids recognized by T-cell receptors. Unlike whole allergens, which can trigger IgE-mediated allergic reactions, T-cell epitopes do not induce IgE-mediated responses. When presented without co-stimulation, peptides can induce T-cell unresponsiveness (anergy) to the whole allergen. Additionally, peptides may induce tolerance by eliminating pathogenic allergen-specific T cells or altering the dominant T-cell phenotype towards Treg cells [116,117,118].

While peptide-based vaccines may have limited impact on humoral antibody responses compared to whole allergen immunotherapy, they offer potential advantages in terms of safety and the ability to modulate T-cell responses. However, it is important to note that while minimizing the risk of IgE-mediated anaphylaxis, peptide-based therapies may still induce T-cell-dependent late-phase asthmatic responses [118].

Several phase 2 studies have evaluated the efficacy of T-cell peptide immunotherapy. Initial studies on T-cell peptide immunotherapy showed promise, with a clinical trial demonstrating reduced rhinoconjunctivitis symptoms in cat-allergic patients treated with a mixture of seven Fel d 1 peptides [119]. However, larger phase 3 field trials involving Fel d 1-derived T-cell peptides (ClinicalTrials.gov, NCT01620762) failed to demonstrate efficacy, leading to the discontinuation of further development for these peptides. These setbacks may be attributed to factors such as a high placebo response in the control group and the inclusion of cat owners who might already have some degree of tolerance due to ongoing exposure to the allergen [2].

Contiguous overlapping peptides are longer sequences of amino acids that encompass a broader range of T-cell epitopes while disrupting IgE epitopes. This approach aims to target T-cell responses without triggering allergic reactions. A phase 2b dose-finding study of birch pollen contiguous overlapping peptides demonstrated a modest but statistically significant treatment effect (7%) over placebo at the highest dose [120,121].

Hydrolyzing whole allergens into medium-chain length peptides can reduce their allergenicity while preserving their ability to induce T- and B-cell responses. This approach may offer a broader range of epitopes compared to synthetic allergen sources [118].

A large clinical trial found that hydrolyzed rye grass allergen peptides administered in a short-term pre-seasonal regimen resulted in a significant reduction in allergy symptoms compared to placebo. The side effects associated with this approach were similar to those observed with conventional SCIT, suggesting that the primary benefit lies in the shortened treatment course [122,123].

### 6.4. B-Cell Peptides

B-cell peptide immunotherapy aims to induce protective humoral antibody responses without stimulating IgE production. One approach involves developing non-IgE-reactive peptides and conjugating them with a carrier protein unrelated to the allergen. This strategy exploits alternative T-helper responses, facilitated by the carrier protein, to induce protective allergen-specific IgG responses while preventing IgE stimulation [17,124].

A placebo-controlled field study involving 181 participants evaluated a mixture of recombinant non-IgE-reactive linear peptides (BM32) derived from grass pollen allergens, fused to a carrier protein (Pre-S protein). The study showed increased levels of allergen-specific IgG1 and IgG4 antibodies with minimal changes in IgE levels. While the primary analysis of combined seasonal symptom medication scores did not reach statistical significance, improvements were observed in asthma symptom scores and quality of life [125,126,127]. The results of phase 3 trials are eagerly awaited to further evaluate the efficacy and safety of this B-cell peptide immunotherapy approach.

### 6.5. DNA-Based Vaccine

DNA-based vaccines have shown promise in mouse models of allergy, inducing preferential TH1 and Treg cell responses while downregulating TH2 cell responses. The repeated use of DNA-based vaccines in humans has raised concerns, including the theoretical risk of plasmid DNA integration into the human genome, the development of anti-DNA antibodies, and the potential for long-term allergen persistence, which could lead to severe allergic reactions [2,16].

A clinical study evaluated a DNA-based vaccine targeting Cry j 2, a major allergen of Japanese cedar pollen allergy. The vaccine incorporated lysosomal-associated membrane protein 1 (LAMP1), a protein that directs the plasmid to the lysosomal compartment, reducing the risk of allergen release and anaphylaxis. After four intramuscular injections, 10 out of 12 participants showed a reduced immediate skin test response to Cry j 2 at 4 months. However, the study did not assess other clinical outcomes [128].

Another approach has involved combining allergens with bacterial DNA sequences containing CpG motifs, which are recognized by TLR-9, a receptor predominantly expressed on human B cells and dendritic cells. A phase 2 trial using a ragweed allergen (Amb a 1) covalently linked to a B-type CpG-containing oligodeoxynucleotide (ODN) showed promising results in ragweed pollen-induced hay fever [86]. However, these findings were not confirmed in a larger phase 3 trial, leading to the discontinuation of this approach [2].

Given their remarkable effectiveness against SARS-CoV-2 during the COVID-19 pandemic, mRNA-based vaccines have garnered significant interest for their potential applications in various medical fields, including allergic diseases [2,16]. Preclinical studies have demonstrated their ability to induce type 1 immune deviation and suppress allergic inflammation in mouse models of allergy [129].

## 7. Passive Immunotherapy

In 1935, Cooke et al. demonstrated that passive transfer of serum from ragweed immunotherapy patients could confer localized protection against ragweed skin prick test reactions in passively sensitized individuals [130]. This passive transfer of immunity was attributed to the transfer of blocking antibodies.

Subsequent studies in cat allergy patients have further validated this concept [131]. A single subcutaneous injection of a mixture of two recombinant anti-Fel d 1 antibodies conferred protection against nasal challenge with whole cat allergen extract for nearly 3 months, accompanied by a reduction in nasal fluid TH2-type cytokines and an increase in serum and nasal IgE-blocking activity [132].

A similar approach has been used to treat seasonal birch pollen allergy, where a cocktail of three monoclonal antibodies targeting the major birch allergen Bet v 1 effectively inhibited the clinical response to birch pollen nasal challenge for at least 2 months [133].

These findings suggest that passive immunotherapy, utilizing monoclonal antibodies targeting specific allergens, may offer a potential treatment option for allergic diseases, particularly for short-term protection or as a bridge therapy during periods of allergen exposure.

## 8. AIT Combination with Biologics

The combination of AIT with biologics, such as monoclonal antibodies targeting specific immune pathways, has emerged as a promising strategy for improving the management of allergic diseases. By combining the disease-modifying effects of AIT with the targeted therapeutic actions of biologics, this approach aims to enhance efficacy and safety while addressing specific challenges associated with AIT [134].

Omalizumab, a humanized monoclonal antibody, targets IgE, preventing it from binding to its receptors and triggering allergic reactions. It is currently approved for the treatment of moderate to severe asthma, chronic rhinosinusitis with nasal polyps, and chronic spontaneous urticaria. When used concurrently with AIT, omalizumab can lessen AIT-associated side effects, increasing tolerability. This allows patients to receive higher doses of allergen more quickly, making AIT suitable for higher-risk patients with asthma and enabling the use of rush protocols [135]. The combination of AIT with omalizumab has demonstrated efficacy in various allergens, including birch, grass, ragweed, perennial allergens, cat, dog, and HDM. Studies have shown a significant reduction in symptom scores, up to 48% less than SCIT alone, with a decrease in rescue medication use during seasonal exposure [136,137].

While the combination of grass pollen subcutaneous immunotherapy and dupilumab, an anti-IL-4 receptor antibody, reduced circulating IL-4-expressing TH2 cells, it did not significantly alter the magnitude or duration of allergen-induced late skin responses compared to allergen immunotherapy alone [138]. A clinical trial (NCT04502966) is currently evaluating the combination of grass pollen allergen with dupilumab, targeting both IL-4-dependent and IL-13-dependent pathways, in the context of inhalant immunotherapy [134].

Tezepelumab, a humanized monoclonal antibody targeting thymic stromal lymphopoietin (TSLP), has demonstrated promise in reducing eosinophilic inflammation, airway hyperresponsiveness, and asthma exacerbations. Additionally, it decreases serum IL-5, IL-13, and total IgE, suggesting potential synergy with AIT. A recent clinical trial investigated the combination of cat dander SCIT with tezepelumab. The study found that tezepelumab combined with SCIT was more effective than SCIT alone at reducing nasal response to cat allergen challenge after 52 weeks. This suppression persisted even one year after stopping treatment, suggesting a potential long-term benefit [139]. These findings suggest that tezepelumab may enhance the effectiveness of AIT by promoting long-term allergen tolerance [134].

Combining AIT with biologics has shown promise in improving short-term efficacy, enhancing safety, and improving patient tolerance during dose escalation. However, further research is needed to assess the long-term benefits and cost-effectiveness of these combination therapies.

## 9. Conclusions

AIT, including SLIT and SCIT, remains a safe and effective treatment for respiratory allergies. While novel approaches, such as the use of adjuvants, recombinant allergens, and biologics, show promise, their clinical efficacy and safety profiles still require further investigation.

Key findings and future directions include:

♦ Traditional AIT: SCIT and SLIT continue to be the primary options for AIT, offering long-term symptom relief and the potential to prevent progression to asthma;
♦ Novel Approaches: While modified allergens combined with novel adjuvants have shown promise in preclinical and early phase clinical trials, their superiority over traditional AIT has not been definitively established;
♦ Personalized Immunotherapy: The development of personalized allergen immunotherapy based on individual sensitization profiles and the identification of specific epitopes, recombinant allergen, offers potential for improved efficacy and safety;
♦ Combination Therapies: Combining AIT with biologics may enhance safety, treatment outcomes and address specific challenges associated with AIT;
♦ Passive Immunotherapy: Recent studies suggest that passive immunotherapy using monoclonal antibodies may offer a viable option for short-term protection against allergic symptoms.

Overall, while AIT remains a valuable treatment option for respiratory allergies, ongoing research is essential to further refine existing approaches and develop novel strategies that can improve efficacy, safety, and patient outcomes.

## Data Availability

The datasets generated during the current study are available from the corresponding author upon reasonable request.

## Abbreviations

AIT	allergen immunotherapy
APC	antigen-presenting cell
AR	allergic rhinitis
Breg	B regulatory cell
CpG	Cytosine-phosphodiester-guanine
DC	dendritic cell
DCreg	dendritic cell-derived regulatory cell
EPIT	epicutaneous immunotherapy
HCP	health care personnel
HDM	house dust mite
Ig	Immunoglobulin
IDIT	Intradermal Immunotherapy
IL	interleukin
ILC2	type 2 innate lymphoid cell
ILCreg	innate lymphoid cell-derived regulatory cell
ILIT	Intralymphatic immunotherapy
LAMP1	lysosomal-associated membrane protein 1
MCT	microcrystalline tyrosine
MPLA	Monophosphoryl lipid A
ODN	oligodeoxynucleotide
pDC	plasmacytoid dendritic cell
SCIT	subcutaneous immunotherapy
SLIT	sublingual immunotherapy
Tfh	T-helper cell
Tfr	T follicular regulatory cell
TGF	transforming growth factor
TH1	T-helper 1 cell
TH2	T-helper 2 cell
TLR	Toll-like receptor
Treg	T regulatory cells
TSLP	thymic stromal lymphopoietin
VLP	virus-like particle
